# The Role of Inhibitory Interneurons in Circuit Assembly and Refinement Across Sensory Cortices

**DOI:** 10.3389/fncir.2022.866999

**Published:** 2022-04-07

**Authors:** Camilo Ferrer, Natalia V. De Marco García

**Affiliations:** Center for Neurogenetics, Brain and Mind Research Institute, Weill Cornell Medicine, New York, NY, United States

**Keywords:** sensory, cortex, critical period, development, interneuron, inhibition, circuit, plasticity

## Abstract

Sensory information is transduced into electrical signals in the periphery by specialized sensory organs, which relay this information to the thalamus and subsequently to cortical primary sensory areas. In the cortex, microcircuits constituted by interconnected pyramidal cells and inhibitory interneurons, distributed throughout the cortical column, form the basic processing units of sensory information underlying sensation. In the mouse, these circuits mature shortly after birth. In the first postnatal week cortical activity is characterized by highly synchronized spontaneous activity. While by the second postnatal week, spontaneous activity desynchronizes and sensory influx increases drastically upon eye opening, as well as with the onset of hearing and active whisking. This influx of sensory stimuli is fundamental for the maturation of functional properties and connectivity in neurons allocated to sensory cortices. In the subsequent developmental period, spanning the first five postnatal weeks, sensory circuits are malleable in response to sensory stimulation in the so-called critical periods. During these critical periods, which vary in timing and duration across sensory areas, perturbations in sensory experience can alter cortical connectivity, leading to long-lasting modifications in sensory processing. The recent advent of intersectional genetics, *in vivo* calcium imaging and single cell transcriptomics has aided the identification of circuit components in emergent networks. Multiple studies in recent years have sought a better understanding of how genetically-defined neuronal subtypes regulate circuit plasticity and maturation during development. In this review, we discuss the current literature focused on postnatal development and critical periods in the primary auditory (A1), visual (V1), and somatosensory (S1) cortices. We compare the developmental trajectory among the three sensory areas with a particular emphasis on interneuron function and the role of inhibitory circuits in cortical development and function.

## Introduction

Sensory information is conveyed from sensory organs in the periphery into modality-specific thalamic nuclei and subsequently into primary sensory cortical regions for further processing. While this basic connectivity is already established at birth, it is subject to extensive activity-dependent refinement during the first and second postnatal weeks in mice, the equivalent of the period spanning from late fetal development through the first few years of postnatal development in humans. At the neocortical level, two main types of neurons constitute the building blocks of cortical circuits: excitatory pyramidal neurons (Pyr) and inhibitory GABAergic interneurons (INs). Although they only constitute ~15%–20% of the neuronal cortical population (Anderson et al., 1997; Cauli et al., 1997; Gonchar and Burkhalter, 1997; Gupta et al., 2000; Kawaguchi, 2001; Butt et al., 2005; Miyoshi et al., 2007; Xu et al., 2010), inhibitory INs are essential for the proper development of cortical circuits (Babij and De Marco Garcia, 2016) and play multiple roles in sensory processing in the mature cortex (Tremblay et al., 2016). GABAergic INs can be classified based on their axonal morphological features and innervation profile in multiple subtypes (DeFelipe et al., 2013; Mihaljevic et al., 2019). While neurochemical expression patterns allow classification of GABAergic INs into three main cardinal classes: Parvalbumin-expressing (PV), Somatostatin-expressing (SST), and the Serotonin 5HT3a Receptor-expressing (5HT3aR) interneurons [which can be subdivided into two main groups: Reelin+ and vasoactive intestinal peptide (VIP) INs (Rudy et al., 2011)], each one with particular properties and functions in cortical processing. In developing mice, inhibitory interneurons originate embryonically in the telencephalon: the Medial Ganglionic Eminence (MGE) and Preoptic Area (POA) generate PV and SST INs; while the Caudal Ganglionic Eminence (CGE) gives rise to 5HT3aR INs (Lim et al., 2018). By birth, INs migrate tangentially into the cortex (Anderson et al., 1997) and other key aspects of interneuron maturation such as laminar distribution, wiring, apoptosis, and circuit refinement take place mainly during postnatal development (Li et al., 2008; Southwell et al., 2012; Lim et al., 2018).

Different sensory cortical areas share the same neuronal composition described above and undergo parallel developmental trajectories. At embryonic stages, thalamocortical axons start invading the developing cortex matching thalamic nuclei with the appropriate cortical regions, for each sensory modality (Sur and Rubenstein, 2005). These thalamocortical projections initially innervate the region under the cortical plate known as the subplate, before reaching their final target thalamo-recipient neurons of layer IV (Kanold and Luhmann, 2010). In rodent S1, thalamic axons are present in the subplate as early as E14, making functional connections with target neurons by E19 (Auladell et al., 2000; Higashi et al., 2002). Subsequently, these axons invade layers V-VI by P0 and make functional synapses onto layer IV around P4 (Agmon et al., 1993). Similar developmental trajectories for thalamocortical innervation occur in V1 (Kanold et al., 2003) and A1 (Viswanathan et al., 2012). However, this process is delayed in A1 in comparison to the other two sensory areas, resulting in a more mature pattern of thalamocortical innervation in V1 and S1 by P5 (Chang et al., 2018). In summary, immature thalamic projections are already present in all three primary sensory areas by birth, undergoing extensive maturation and rearrangement during the first two postnatal weeks.

During the first postnatal week in mice, cortical activity is dominated by spontaneous waves of activity (Ackman et al., 2012; Babola et al., 2018; Che et al., 2018; Iannone and De Marco Garcia, 2021). This early synchronized spontaneous activity is essential for the proper maturation of cortical circuits and thought to be the substrate for the development of mature connectivity (Leighton and Lohmann, 2016). A characteristic landmark of the mature cortex is the presence of cortico-cortical connectivity, which develops postnatally. One of the main cortico-cortical projections connects the two hemispheres through callosal axons, in a homotypic fashion. In both S1 and V1, callosal axons cross the midline by P3 and display extensive arborization into the contralateral cortex during the second postnatal week (Mizuno et al., 2007; Wang et al., 2007). This connectivity develops in an activity dependent manner (Wang et al., 2007), being therefore influenced by early spontaneous activity of the maturing cortex.

By the second postnatal week, sensory input increases upon the onset of active whisking, ear and eye opening. Simultaneously, cortical activity in primary sensory areas desynchronizes (Golshani et al., 2009; Rochefort et al., 2009; Clancy et al., 2015; Che et al., 2018; Luhmann and Khazipov, 2018) allowing proper coding of sensory information. Shortly after the second postnatal week, cortical circuits are highly plastic and can be modified by sensory experience during developmental windows known as critical periods (CP; Hensch, 2005). Recent experimental advances in genetic targeting, *in vivo* imaging and transcriptomics have demonstrated the crucial role of inhibitory INs in the proper function of developing cortical circuits.

In this review, we will summarize the developmental trajectories of cortical activity, sensory onset and critical periods in the primary sensory areas A1, V1, and S1. Namely, we will compare the timing and known mechanisms during three main epochs of cortical development: (1) the presence of synchronized spontaneous cortical activity early in development; (2) a period of desynchronization and the onset of sensory-evoked responses; and (3) critical periods of experience-dependent cortical plasticity. We emphasize the developmental maturation of the different IN subtypes and their diverse roles in cortical function spanning from the first postnatal week, up until the critical period in primary cortical sensory areas. In addition, we discuss current litterature regarding the cellular and molecular mechanisms underlying critical period plasticity through direct and indirect regulation of PV INs in the three sensory areas.

## Development of Neuronal Activity and Cortical Microcircuits in The Auditory Cortex

The auditory system conveys acoustic information from the ears to the brain in multiple relay areas for sensory processing. Auditory information is initially transduced by hair cells in the cochlea, and subsequently transmitted to brainstem and midbrain auditory structures. Then, sensory input is relayed into the auditory thalamus (Medial Geniculate Body, MGB) and finally reaches the primary auditory cortex (A1) for further processing. In the mature cortex, auditory information is conveyed and organized in A1 in such a way that different sound frequencies are spatially segregated in a tonotopic fashion (Brewer and Barton, 2016).

### Early Synchronized Activity

**Figure 1:**
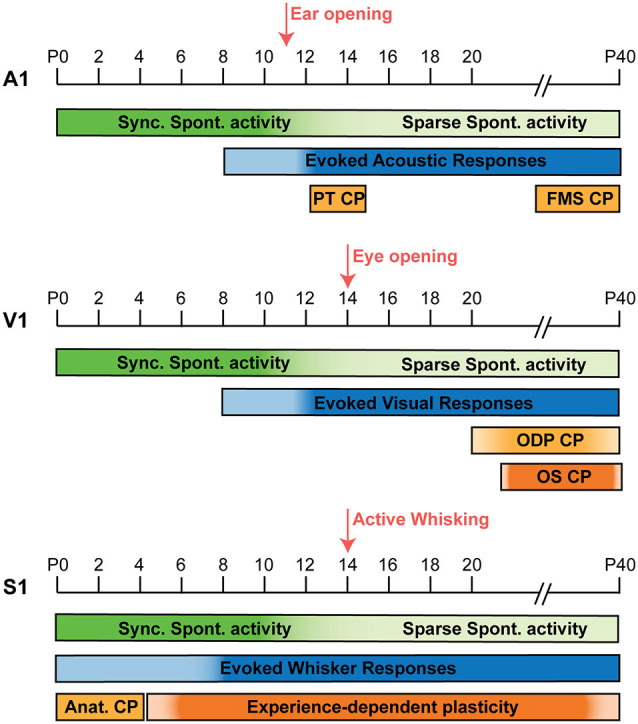
Timeline comparison of cortical activity development in A1, V1, and S1. Development of cortical activity in mice from P0 to P40 in A1, V1, and S1. In the three areas, synchronized spontaneous activity is present during the first postnatal week, but it desynchronizes through the second postnatal week in A1 (Babola et al., 2018; Meng et al., 2020); V1 (Rochefort et al., 2009; Ackman et al., 2012; Siegel et al., 2012), and S1 (Golshani et al., 2009; Che et al., 2018). In A1 (top panel), the onset of evoked sensory activity takes place by the end of the first postnatal week and it becomes more prominent upon ear opening at P11 (Anthwal and Thompson, 2016; Makarov et al., 2021). Two auditory critical periods for pure tones (PT CP; Barkat et al., 2011) and Frequency modulated sweeps (FMS CP; Bhumika et al., 2020) take place shortly after, P12–15 and P31–38 respectively. In V1 (middle panel), evoked visual responses start by the end of the first postnatal week and become more reliable by the second postnatal week (Colonnese et al., 2010), around the time of eye opening (~P14). The critical period for ocular dominance (ODP CP) takes place between P21 and P35 (Hensch, 2005; Espinosa and Stryker, 2012) and an orientation selectivity (OS) CP has been shown in google-reared mice between P28 and P49 (Yoshida et al., 2012). In S1 (bottom panel), passive whisker stimulation can trigger evoked responses from birth (Anton-Bolanos et al., 2019), but these responses become more reliable around P6–8 (Colonnese et al., 2010) and active whisking onset occurs around P14 (Landers and Philip Zeigler, 2006). An early anatomical CP is observed in S1 from P0 to P4, capable of altering barrel formation and thalamocortical innervation (Durham and Woolsey, 1984; Lee et al., 2009). While throughout life, different paradigms of sensory deprivation can induce discrete forms experience-dependent plasticity in particular layers/synapses (Nowicka et al., 2009; Wen and Barth, 2011; Gainey et al., 2018).

The development of cortical auditory representations is a protracted process, regulated by both spontaneous and sensory evoked actvities. In mice, the ears are closed perinatally and the auditory system is shaped by spontaneous activity in the absence of sensory-evoked stimuli (Figure 1, top panel; Wang and Bergles, 2015). From postnatal day (P) 0 to P10 cochlear cells in the inner ear are spontaneously active (Tritsch and Bergles, 2010) and as early as P7 spontaneous activity has been recorded *in vivo* in the inferior colliculus and A1 (Babola et al., 2018; Meng et al., 2021). Before hearing onset, spontaneous activity in the cortex and midbrain originates from the cochlea (Tritsch et al., 2010) and follows a tonotopic organization reminiscent of the mature auditory system (Babola et al., 2018). Thus, cortical activity in A1 is purely spontaneous in nature in the first 10 days of postnatal development.

### Onset of Sensory-Evoked Responses and Desynchronization

Hearing onset in mice occurs around P11, upon ear canal opening (Anthwal and Thompson, 2016). However, high-intensity, bone-conducted acoustic stimuli can evoke cortical responses in A1 as early as P8 (Makarov et al., 2021). While the subcortical connectivity is already present at this stage, cortical connectivity is still undeveloped and progressively matures in response to the acoustic sensory environment. By this time, thalamocortical connectivity transitions from innervation of the subplate to innervation of layer IV (Kanold and Luhmann, 2010; Barkat et al., 2011), allowing robust sensory-evoked activation of A1. Furthermore, *in vivo* 2-photon calcium imaging experiments have shown that spontaneous pairwise correlation in layers II-III decreases while sensory-evoked correlation increases peaking at P15 (Meng et al., 2020), indicating sparsification of spontaneous activity and strengthening of thalamic driven sensory responses during the second postnatal week.

### Critical Period Plasticity

Shortly after ear opening, between P12 and P15, the tonotopic organization of the auditory cortex can be modified by the presence or absence of auditory stimulation during the CP (Figure 1, top panel). Presentation of a pure tone (PT), only during the CP, expands the best frequency tonotopic representation in A1 towards the exposed tone by changing the topography of thalamocortical inputs onto layer IV (Barkat et al., 2011). Similar tonotopic plasticity has been observed in rats (Zhang et al., 2002; de Villers-Sidani et al., 2007; Keuroghlian and Knudsen, 2007) and leads to behavioral deficits in perception of the overrepresented frequency (Han et al., 2007). Other sound features have different critical periods, as it is the case of the CP for frequency modulated sweeps (FMS), in which exposure to these complex sounds during a defined developmental window between P31 and 38 alters frequency representations in A1 (Bhumika et al., 2020). Moreover, long-term rearing of mice in the presence of white noise delays the CP for pure tones but not for FMS (Nakamura et al., 2020), indicating independent substrates for the two critical periods. These studies demonstrate the existence of different critical windows for acoustic stimuli in A1, however, the specific differences and underlying mechanisms remain unclear.

### Cortical Circuit Maturation and the Role of GABAergic Interneurons Across Development

Local and long-range connectivity in A1 during the first postnatal weeks, undergoes extensive changes, leading to the mature circuit configurations present in the adult. In regards of local connectivity, laser-photostimulation experiments have shown that local glutamatergic responses onto LIV-V INs transition from silent (NMDAR-only) synapses at P6 to AMPAR-containing translaminar synapses at P13 (Deng et al., 2017). Less is known about the precise development of long-range connectivity in A1. However, the presence of functional thalamocortical connectivity has been observed at P7 (Babola et al., 2018) and retrograde labeling experiments have shown the presence of corticocollicular connections mediated by layer V neurons by P5 (Chang et al., 2018). In subsequent development, LII-III Pyr receive transient heightened subgranular (LV-VI) excitatory input during the peak of the CP for pure tones, which subsequently decreases and gets restricted to LIV/II-III in mature A1 (Meng et al., 2020). It is unclear if this transient cortical circuit constitutes a substrate for critical period plasticity.

While details on the maturation of specific circuit patterns are just emerging, some of the evidence in adult A1 highlights the importance of inhibitory connectivity and could be indicative of its potential role during development. In adult mice, LI Reelin+, 5HT3aR+ INs which also express Neuron Derived Neurotrophic Factor (NDNF) receive top-down inputs from the higher order medial geniculate nucleus as well as cholinergic innervation from the nucleus basalis and in turn inhibit distal dendrites of LII-III Pyr, the strength of this inhibition serves as the substrate for acoustic memory coding in a fear conditioning paradigm (Abs et al., 2018; Pardi et al., 2020). In contrast, SST inhibitory output onto both LII-III Pyr and NDNF INs does not change in response to fear conditioning, but can override NDNF-mediated inhibition upon strong sensory stimulation (Abs et al., 2018). Therefore, SST and NDNF inhibitory circuits result in two different patterns of LII-III Pyr inhibition dependent on the learning task and the strength of sensory stimulation, respectively. On the other hand, LI IN-mediated PV inhibition and subsequent Pyr disinhibition in LII-III, driven by cholinergic innervation to LI, mediates fear conditioning and learning of sound-shock association (Letzkus et al., 2011). In contrast, PV INs in LV are engaged by callosal, contralateral A1 projections, which in turn primarily inhibit cortico-cortical Pyr projecting neurons and to a lesser extent cortico-colicular Pyr (Rock and Apicella, 2015), mediating interhemispheric connectivity and bilateral integration of acoustic stimuli. All this evidence shows that mature cortical inhibitory circuits in A1 act as switches regulating different paths for sensory information flow and auditory processing in response to learning rules, stimulus features, and the internal state of the animal.

## Development of Neuronal Activity and Cortical Microcircuits in The Visual Cortex

Similar to the auditory system, the visual system develops in an activity-dependent fashion, relying first solely on spontaneous and subsequently light-evoked responses for its development. Photosensitive cells in the retina transduce light, converting it into an electrical signal that travels *via* the optic nerve into the visual thalamus (Lateral Geniculate Nucleus, LGN), which then sends projections into V1 for central processing. Topographic representation of stimulus features is a conserved feature across sensory systems. The visual cortex displays retinotopic organization such that specific regions of the retina are represented in particular regions of V1. The basic organization and connectivity of the visual system is present in mice shortly after birth; however, it undergoes extensive tuning and reorganization during the first few postnatal weeks.

### Early Synchronized Activity

During the first postnatal week, spontaneous activity (Figure 1, middle panel) regulates the emergence of subcortical and cortical circuits. At this stage, the retina itself generates different types of spontaneous activity, playing an instructive role in the development and organization of the visual pathways (Torborg and Feller, 2005). Synchronized spontaneous activity can be recorded *in vivo* in V1 as early as P3 in mice (Ackman et al., 2012; Siegel et al., 2012). Cortical activity at this stage can be classified in: L (Low synchronicity)-events, which compromise 20%–80% of coactive neurons and are reduced by bilateral enucleation; and H (High synchronicity)-events, consisting of ≥80% of coactive neurons and are generated independently of retinal activity, instead mediated by gap-junctions (Siegel et al., 2012). During the first postnatal week L-type events are twice as frequent (1/min) compared to H-events (Leighton et al., 2021). By eye opening, L-type event frequency triples, becoming the dominant form of activity at this stage, while H-event frequency remains the same (Siegel et al., 2012). The highly synchronous nature of H-events can sustain homeostatic regulation of synaptic strength (Turrigiano and Nelson, 2004). On the other hand, L-type activity is relatively sparse, resembles visually-evoked responses after eye opening (Ohki et al., 2005) and has been proposed to mediate retinal topographic organization. This is evidenced by the activity-dependent refinement of thalamocortical innervation during the first postnatal week, such that desynchronization of retinal activity and L-type event disruption, results in diffuse thalamocortical innervation and imprecise retinotopic organization of cortical activity in V1 (Cang et al., 2005).

### Onset of Sensory-Evoked Responses and Desynchronization

The end of the first postnatal week marks the onset of sensory-evoked responses (Figure 1, middle panel) such that illumination of the eye, which is still closed, evokes bursting activity in V1, but only by P12 these responses become more consistent and reliable (Colonnese et al., 2010). By P14, with the onset of eye opening, spontaneous activity desynchronizes and becomes more alike the adult state. At the same time, other mature features of V1 including retinotopic organization, eye-specific segregation, and orientation tuning are already present (Smith and Trachtenberg, 2007; Rochefort et al., 2009; Ko et al., 2013).

### Critical Period Plasticity

Beyond the second postnatal week, during a developmentally defined period between P21 and P35 (Figure 1, middle panel), cortical visual responses are sensitive to the presence/absence of sensory input during the Critical Period for Ocular Dominance Plasticity (ODP; Hensch, 2005; Espinosa and Stryker, 2012). In normal conditions, cortical responses are lateralized, meaning most neurons in V1 are responsive to stimulation of the contralateral eye and less so to ipsilateral stimulation. However, during the critical period for ODP, monocular deprivation can shift this bias, increasing cortical responses to the ipsilateral (open) eye and reducing responsiveness to the contralateral (deprived) eye, while the same manipulation either before or after the critical period has no effect on eye dominance (Gordon and Stryker, 1996). In monocular deprivation experiments, ODP is accompanied by a reduction in the strength of thalamocortical innervation to layer IV (Wang et al., 2013). Accompanied also by intracortical changes, decreasing layer IV Pyr firing, through enhanced inhibition, an effect that is only observed during the CP (Maffei et al., 2004, 2006). These changes reconfigure the cortical circuit to be less responsive to the deprived eye and subsequently increase responsivity to the spared eye. ODP is linked to the maturation of GABAergic signaling (Fagiolini and Hensch, 2000; Fagiolini et al., 2004) and in particular PV IN maturation (Kuhlman et al., 2013; Hooks and Chen, 2020).

In comparison to ocular dominance, classical paradigms of sensory deprivation have little to no effect in the development of other features of the visual response in mice, such as orientation and direction selectivity. Both orientation (OS) and direction (DS) selectivity in neurons are present upon eye-opening and their responses remain unchanged in animals dark-reared up until P30 (Rochefort et al., 2011). While a different manipulation, rearing animals with lenses to restrict contours to only one orientation (O’Hashi et al., 2007), results in overrepresentation of the exposed orientation in layers II/III neurons (Kreile et al., 2011). This critical period for orientation selectivity plasticity (OS CP) in goggle-reared mice occurs between 4 and 7 postnatal weeks, with a lower degree of plasticity remaining into adulthood (Yoshida et al., 2012). These studies prove the existence of distinct experience-dependent critical periods for ocular dominance and orientation selectivity in mice, whether OS plasticity shares the same mechanisms as ODP remains unknown.

CP plasticity has also been characterized in carnivore species, finding both similarities and differences to rodents. Alike the mouse, a defined critical period for ODP has been identified in both cats and ferrets using monocular deprivation (Albus and Wolf, 1984; Issa et al., 1999). OS is also present in both species by eye-opening (Roy et al., 2018) and remains unchanged in dark-reared animals (Van Hooser et al., 2012) but google-rearing paradigms can also induce OS CP plasticity in cats (Tanaka et al., 2009). In contrast to mice, direction selectivity in ferrets is not present at the time of eye opening and dark-rearing prevents DS acquisition (Li et al., 2006). Thus, although similar forms of visual plasticity are present across species, some forms of experience-dependent plasticity, such as DS plasticity, are species-specific. This specificity might represent differences in the organization or development of the visual system in different animal groups.

### Cortical Circuit Maturation and the Role of GABAergic Interneurons Across Development

Recent studies have shown that transient developmental circuits involving inhibitory INs are instructive for proper cortical maturation. An example of this is the early thalamic innervation of LI NDNF+ interneurons by the second postnatal week, which is required for the subsequent strengthening of cortico-cortical inputs observed in this group of mature INs (Ibrahim et al., 2021). Similarly, by the beginning of the second postnatal week SST INs restrict the spread and number of cells participating in spontaneous L-events (Leighton et al., 2021) and may act by preserving retinal topography and circuit plasticity before PV IN maturation. Cortical activity also influences IN maturation during this time: Chandelier cells, a subtype of axon-targeting PV INs, undergo an active process of apoptosis driven by retinal as well as callosal contralateral activity in the binocular V1 (Wang et al., 2021), this activity-dependent apoptosis is required for normal binocular vision in mature animals. Thus, even at immature stages inhibitory circuits play an important role on visual cortical function and maturation.

Multiple studies have shown the important and diverse roles of cortical INs in the mature visual system. The canonical patterns of inhibitory connectivity have been established in adult V1: PV INs strongly inhibit Pyr and one another; SST INs preferentially inhibit Pyr and 5HT3aR INs; while VIP INs play a disinhibitory role, predominantly targeting SST cells (Pfeffer et al., 2013). The interplay of these local circuit patterns and the selective engagement of different IN subtypes by select afferents enrich the cortical computational power, necessary for visual processing. PV INs are engaged in cortico-cortical bottom-up feedforward inhibition (from V1 to high-order visual areas) as well as feedback top-down innervation (from high-order visual areas to V1). While LI INs are engaged by top-down projections from secondary visual, as well as higher order cognitive areas (Gonchar and Burkhalter, 2003; Ibrahim et al., 2021). Along these lines, SST cells are preferentially excited by horizontal axons and therefore engaged by stimulation of the receptive field surround, contributing to Pyr surround suppression (Adesnik et al., 2012). In addition, VIP-SST circuits are necessary for context modulation of visual stimuli (Keller et al., 2020): upon presentation of two concentric visual stimuli with varying degrees of similarity, if the stimuli center and surround are similar VIP INs are inactive while SST INs actively inhibit Pyr; however, if the center and surround are different the VIP-SST disinhibitory loop gets engaged, resulting in increased Pyr activity (Keller et al., 2020). On the other hand, PV INs, given their extensive innervation of Pyr and self-inhibition, modulate the gain of cortical circuits, without affecting the orientation tuning properties of Pyr neurons (Atallah et al., 2012). These select examples, highlight the relevance of inhibitory microcircuits in visual cortical processing in adult V1.

## Development of Neuronal Activity and Cortical Microcircuits in The Somatosensory Cortex

The barrel cortex is a specialized part of the rodent somatosensory cortex receiving tactile information from the whiskers. Due to its recognizable structure and ease of manipulation, it has been used as a model of sensory cortical processing, therefore we will focus on this specific part of S1 for further discussion. Following a developmental sequence, maturation of somatosensory circuits in response to intrinsic and sensory-evoked activity begins earlier than that of the auditory and visual systems (Figure 1, bottom panel). In rodents, tactile sensory information is transduced by mechanoreceptors located at the base of the whiskers and activated by their movement. This information travels as action potentials through the trigeminal nerve into the brainstem, which then sends projections to the thalamus. Two main thalamic areas relay somatosensory information to the cortex: the ventral posterior medial (VPM) nucleus and the posterior medial (POm) nucleus sending thalamocortical projections to layer IV and layers I/V, respectively. In S1, a specialized region known as the barrel cortex receives whisker-selective thalamic projections, forming anatomical and functional structures, in such a way that each whisker’s receptive field is represented in a particular region of the cortex known as a barrel. After primary sensory processing in S1, connectivity with motor cortex (M1), contralateral S1 (cS1), and secondary somatosensory cortex (S2) allows further processing of sensory information. Similar to what we described previously for A1 and V1, the basic connectivity of S1 is present shortly after birth, however, it undergoes extensive maturation driven by the interplay between spontaneous and sensory-evoked activity during the first postnatal weeks.

### Early Synchronized Activity

Spontaneous activity is present throughout the somatosensory system in early development (Figure 1, bottom panel). Seminal studies *in vitro* have shown different types of spontaneous activity in developing S1: cortical early network oscillations (cENOs) and spontaneous plateau assemblies (cSPAs), dependent on glutamatergic activity and gap junctions respectively (Allene et al., 2008), are observed upon birth and for a few days; while giant depolarizing potentials (GDPs), relying on both GABAergic and glutamatergic activity (Allene et al., 2008), are observed by the end of the first and into the second postnatal week. These observations have been validated *in vivo* using calcium imaging in un-anesthetized mice. Spontaneous thalamic activity in the VPM has been recorded as early as embryonic day (E) 17.5 (Moreno-Juan et al., 2017). These waves reach the cortical plate and activate broad regions of the developing cortex and is it not until birth (between P0 and P4) that cortical activity becomes spatially restricted into a protomap of the barrel cortex (Anton-Bolanos et al., 2019).

### Onset of Sensory-Evoked Responses and Desynchronization

**Figure 2:**
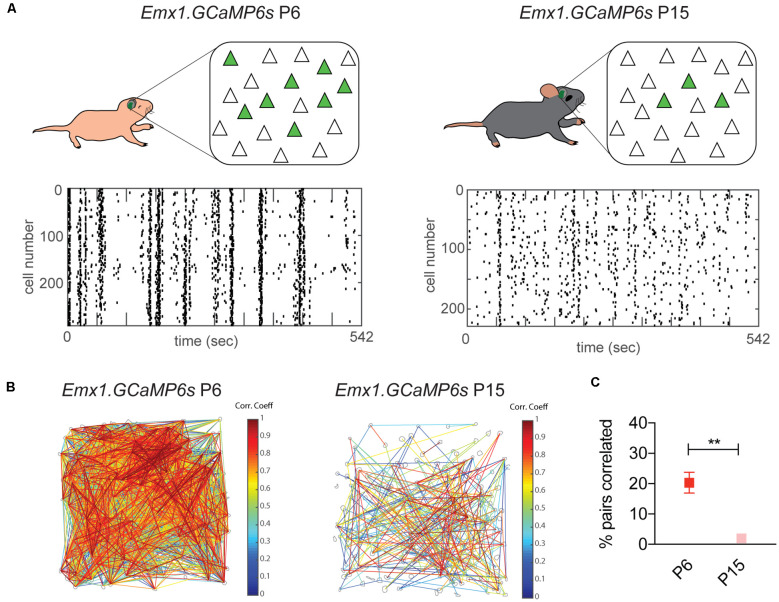
Sparsification of spontaneous cortical activity in S1 by the end of the second postnatal week. Desynchronization of spontaneous cortical activity in S1 measured using *in vivo* calcium imaging. **(A)** Top panel: diagrams of *in vivo* imaging of Emx1.GCaMP6 s mice at P6 and P15, with cranial windows placed over S1. The number of spontaneously co-active Pyr neurons (green) at any given time decreases between P6 and P15. Bottom panel: representative raster plots of neuronal calcium activity at both ages. Each row represents a single neuron imaged during 542 s. Each tick represents the onset of a single calcium event. At P6 neuronal activity is characterized by the co-activation of most neurons imaged in the field of view, visualized in vertical arrangements in the raster plot, while at P15 neuronal activity becomes less synchronized resulting in a “salt and pepper” pattern in the raster plot. **(B)** Visualization of correlated neuronal activity corresponding to recordings in **(A)**. Gray contours indicate detected somas in which calcium signals were analyzed. Significantly correlated cell pairs are connected by lines. Line color indicates the magnitude of the correlation coefficient of the connecting pair. **(C)** Percentage of pairs that are significantly correlated decreases from P6 to P15. Unpaired t-test, **p = 0.0027. Derived with permission from Che et al. (2018).

While similar in many ways, the maturation profile of developing S1 is accelerated in comparison to other sensory cortices (Figure 1). In contrast to the later onset of sensory-evoked responses in A1 and V1 by the end of the first postnatal week, passive whisker stimulation can evoke responses in S1 even before birth (Anton-Bolanos et al., 2019). However, sensory-evoked responses do not become reliable until P6–8 in S1, while the same process does not occur in V1 until P12 (Colonnese et al., 2010; Che et al., 2018). Therefore, neuronal activity in S1 during the first postnatal week is characterized by an overlap of both synchronized spontaneous activity and sensory-evoked responses. This overlap lasts until the end of the second postnatal week, when spontaneous neuronal activity in S1 desynchronizes (Figure 2; Golshani et al., 2009). Using *in vivo* imaging in un-anesthetized mice (Figure 2A), this developmental desynchronization is evidenced by a reduction in the % of Pyr pairs correlated from P6 to P15 (Figures 2B,C; Che et al., 2018). While this occurs in a period coincident with the onset of active whisking at ~P14 (Landers and Philip Zeigler, 2006), the sparsification of Pyr neuron activity is independent of sensory input (Golshani et al., 2009). These developmental changes result in a mature state, characterized by sparse neuronal activity and dominated by sensory-evoked responses. How the interplay between sensory-evoked and spontaneous activity promote cortical maturation in S1 is still poorly understood and a matter of active research.

### Critical Period Plasticity

In addition to the accelerated maturation of S1, another particularity of this area is the difficulty in defining an equivalent critical period to ODP or the CP for pure tones, described above for V1 and A1 respectively. Early studies identified a structural critical period from P0 to P4 for barrel formation (Durham and Woolsey, 1984). Using trigeminal nerve transection, these studies showed that the barrels in LIV disappear and the presence of aberrant thalamocortical innervation (Belford and Killackey, 1980; Catalano et al., 1995). However, this approach not only causes sensory deprivation, but a permanent nerve damage that could contribute to the observed phenotype. In contrast, a more subtle experimental approach, using bilateral whisker trimming from P0 to P3 and then allowing whisker regrowth and testing anatomical and behavioral changes at P30, results in a milder phenotype, increasing dendritic span and the number of spines of LIV stellate cells with accompanying long-lasting behavioral defects (Lee et al., 2009). While another study, using a similar manipulation, trimming all but one or two whiskers from P0 to P96, showed a similar reduction in VPM innervation independently of the age tested and eventual reversion of this effect upon whisker regrowth (Wimmer et al., 2010).

On top of the structural changes, whisker trimming also causes functional neuronal changes at later developmental stages and throughout life. While LIV to LII-III excitatory inputs display a discrete sensory-dependent sensitive period from P12 to P14, intra LII-III connectivity plastic period is slightly delayed in comparison, taking place from P13 to P16 (Wen and Barth, 2011). And sustained single-row whisker trimming from P7 onwards, causes two phases of inhibitory weakening onto LIV neurons, a transient one at P15 and a second sustained period from P22 to P30 (Gainey et al., 2016). On the other hand, electrical stimulation in brain slices can induce long-term potentiation of thalamocortical inputs to layer IV during a critical period for plasticity from P3 to P7 (Crair and Malenka, 1995). Thus, with the caveat of the differences in the experimental manipulations, these studies indicate a higher degree of plasticity and a less-well defined critical period in S1 (Figure 1, bottom panel), with different timings for specific layers/synapses. Since whiskers can naturally fall off and regrow multiple times throughout a mouse lifetime, it is not surprising to find life-long forms of sensory experience-dependent plasticity in the rodent somatosensory system and a higher degree of plasticity compared to the visual and auditory cortices.

### Cortical Circuit Maturation and the Role of GABAergic Interneurons Across Development

Transient developmental circuits help molding cortical activity and the maturation of S1. From the first to the second postnatal week, there is an increase in the strength of LIV to LII-III glutamatergic input, as well as the emergence of LII-III to LV inputs, which transition from silent (NMDAR only) to AMPAR-mediated synapses (Anastasiades and Butt, 2012). Similarly, LII-III glutamatergic input to LIV immature fast-spiking INs (putative PV) reduces over development, resulting in only local LIV innervation; while LIV non fast-spiking (putative SST) receive both LII-III and LIV excitatory inputs all along the same developmental time (Anastasiades et al., 2016). These changes help shape cortical activity into a mature state, necessary for sensory processing in adult animals.

Maturing interneurons also play pivotal roles during the first few postnatal weeks. SST INs in LVb receive direct thalamic innervation by the end of the first postnatal week and inhibit LIV stellate cells. Blockage of this transient inhibition delays thalamocortical innervation of LIV, which normally occurs by the end of the second postnatal week (Marques-Smith et al., 2016). In addition, MGE-derived interneurons (both PV and SST) participate and help restrict spontaneous activity by the end of the first postnatal week (Duan et al., 2020). In LI, thalamic innervation develops by the first postnatal week (Galazo et al., 2008), targeting Reelin INs and regulating barrel map formation (Che et al., 2018). Thus, INs play an active role regulating neuronal activity in S1 during the first postnatal week. Later on, during the second postnatal week, both SST and VIP INs respond more to multi-whisker compared to single-whisker stimulation; while by the third postnatal week, SST responses remain the same but VIP INs lose this distinction, due to a developmental reduction in direct thalamic innervation onto VIP INs (Kastli et al., 2020). And similarly, chandelier cells (ChC) form synapses between P12 and P18, at which stage GABA release in the AIS is depolarizing, promoting neuronal activity (Pan-Vazquez et al., 2020). However, in the adult ChC activation decreases Pyr activity as GABA becomes hyperpolarizing in the AIS (Pan-Vazquez et al., 2020). These findings illustrate the progressive involvement and functional changes of inhibitory circuits in stimuli representation at different stages of development.

The different GABAergic IN subtypes also play multiple roles in adult S1. PV INs receive strong excitatory input from the thalamus (Sermet et al., 2019), contralateral S1 and S2 (Naskar et al., 2021), mediating feedforward Pyr inhibition and regulating thalamic and inter-hemispheric responses. On the other hand, M1 primarily engages VIP INs (Naskar et al., 2021), which increase Pyr activity by means of the VIP-SST disinhibitory circuit (Lee et al., 2013). This same circuit regulates LII-III SST activity, silencing these neurons during active whisking (Munoz et al., 2017). In contrast, LIV SST INs are activated by active whisking due to scarce VIP to SST innervation in this layer (Munoz et al., 2017), showing how the same IN subtype can also play differential roles depending on their laminar allocation. Lastly, LI INs contribute to precise stimulus-evoked responses *via* lateral inhibition (Fan et al., 2020) and therefore participating in single whisker discrimination. All this evidence highlights the prominent role of inhibitory microcircuits in the regulation of cortical network dynamics.

## Common Principles for Critical Period Plasticity Across Sensory Cortices

**Figure 3:**
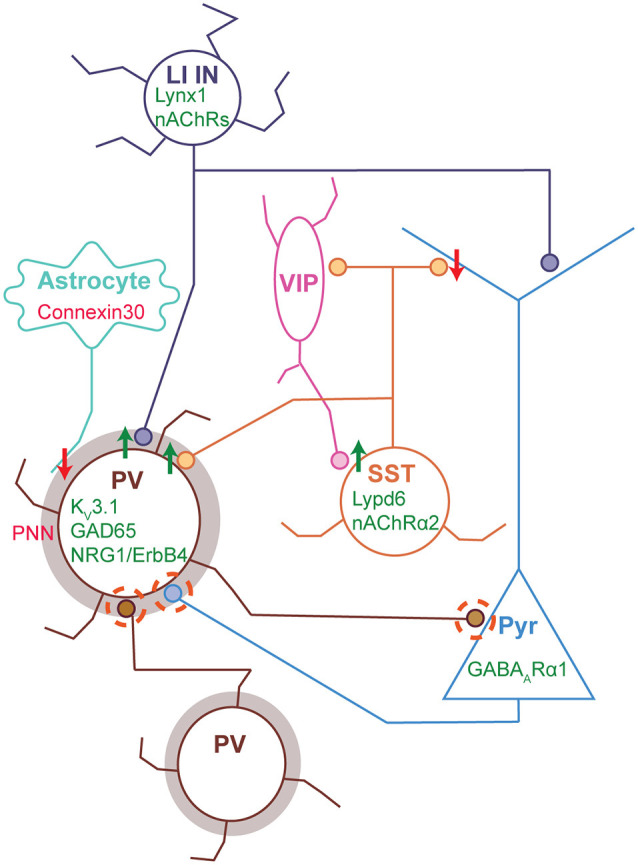
Molecular and synaptic loci of plasticity during sensory critical periods. Cortical circuit schematic with major synaptic connections and molecular factors regulating critical period plasticity. The circuit depicted shows the circuit motifs and cellular components regulating CP plasticity: basket PV INs (Brown) inhibit one another and exert strong inhibitory control of Pyr (light blue) through perisomatic inhibition; layer I INs (dark blue) inhibit the apical dendrites of Pyr and can also target PV cells, resulting in simultaneous Pyr somatic disinhibition and dendritic inhibition; Martinoti SST INs (yellow) across layers target Pyr apical dendrites and also receive input from VIP INs (pink), such that VIP activation results in Pyr disinhibition; while a subset layer IV SST INs target PV INs preferentially, resulting in disinhibition; and astrocytes regulate the extracellular matrix and PNN formation onto PV INs. PV IN maturation determines both the onset and closure of cortical critical periods. Maturation of PV intrinsic properties, synaptic inputs (both excitatory and inhibitory, dashed circles), and inhibitory synaptic output onto Pyr (dashed circle) are all crucial for CP plasticity. Expression of K_V_3.1, GAD65 and NRG1/ErbB4 in PV INs promote (green) normal CP plasticity (Hensch et al., 1998; Matsuda et al., 2021; Zheng et al., 2021), while PNN (brown shadow) maturation in PV INs prevent and close CP plasticity (Pizzorusso et al., 2002; Nowicka et al., 2009; Sigal et al., 2019). In addition, GABA_A_α1 receptor expression in Pyr (in putative PV synapses) is necessary for CP plasticity (Fagiolini et al., 2004). Both SST and LI INs can induce CP plasticity indirectly by means of PV IN inhibition, such that the expression of molecular factors promoting SST (Lypd6 and nAChRα2) or LI IN (Lynx1 and nAChRs) activity enhance plasticity (Takesian et al., 2018; Sadahiro et al., 2020). On the other hand, VIP IN-mediated Pyr disinhibition, *via* SST inhibition, also promotes cortical plasticity (Fu et al., 2015). In contrast, connexin 30 expression in astrocytes restricts CP plasticity *via* PNN maturation in PV INs (Ribot et al., 2021). Green font/arrows represent molecules or synapses promoting CP plasticity, while red font/arrows represent those preventing plasticity. Abbreviations: PV, Parvalbumin; Pyr, Pyramidal cell; SST, Somatostatin; LI INs, Layer I interneurons; VIP, Vasoactive intestinal peptide; PNN, Perineuronal net; nAChRs, Nicotinic Acetylcholine receptors; NRG1, Neuregulin 1; K_V_3.1, Potassium channel 3.1; GAD65, Glutamic acid decarboxylase 65-kilodalton.

How does sensory experience influence cortical development and what determines the specific timing of critical period plasticity? These questions have been extensively studied in the visual system in the context of ODP, as this phenomenon was initially observed in this area; however, some common principles of sensory critical periods have emerged across sensory cortices, involving cortical inhibition in this process (Figure 3). Among other factors, PV interneurons are key players determining the timing and plasticity of sensory critical periods. In this section, we will describe some of the initial and recent evidence linking inhibitory interneurons to sensory critical periods and some of the known mechanisms involved. More detailed discussions about other possible molecular and cellular mechanisms regulating critical period plasticity have been summarized previously (Levelt and Hubener, 2012).

In the last two decades, seminal studies in V1 implicated the strength of the inhibitory tone to the precise timing of the critical period for ODP. Removal of the synaptic GABA synthetizing enzyme GAD65 delays the onset of the critical period indefinitely, however plasticity can be restored in this model upon infusion of the GABAR agonist diazepam (Hensch et al., 1998). While early exposure to diazepam induces a premature CP for PT in A1 (Nakamura et al., 2020) and ODP in V1 (Fagiolini et al., 2004), by enhancing GABAergic activity. But this effect in V1 is dependent on the action of α1 GABA_A_Rs (Fagiolini et al., 2004), predominantly enriched at synapses receiving input from PV INs (Klausberger et al., 2002). Moreover, in the context of the auditory system, chemogenetic silencing of PV activity can reopen CP plasticity in adult A1 (Cisneros-Franco and de Villers-Sidani, 2019). Subsequent studies have delved deeper into the mechanisms by which PV INs can control the timing of critical period plasticity. PV interneuron maturation occurs in parallel with the timing of the critical period for ODP in V1: PV expression does not start until the second postnatal week and the intrinsic and synaptic properties of PV INs mature extensively during the second and third postnatal weeks (Lazarus and Huang, 2011; Akgul and Wollmuth, 2013; Helm et al., 2013; Ferrer et al., 2018). Moreover, PV inhibitory output onto Pyr strengthens upon eye opening, while SST to Pyr connections weaken during the same period (Guan et al., 2017). Studies using transplantation of embryonic MGE-derived interneurons (future PV and SST) in adult animals demonstrated that this manipulation reopens ODP in adult mice, with the timing equivalent to what the CP would have been in the donor (Davis et al., 2015). However, the loci of this plasticity are the host PV INs, which interact with the donor INs increasing NRG1/ErbB4 signaling, regaining CP plasticity (Zheng et al., 2021). Additionally, the relative timing of the different critical periods across sensory areas (Figure 1; first in S1, then in A1 and last in V1), has been linked to the timing of PV IN maturation, as it occurs in the same order along the mouse cortex (del Rio et al., 1994; Reh et al., 2020). These studies demonstrate the importance of PV IN maturation on establishing the timing of critical period plasticity.

Other molecules associated to PV inhibitory function and maturation can influence the CP timing (Figure 3). The extracellular matrix of PV INs, forms a net of proteoglycans around the soma and proximal dendrites and perforated by synapses, known as perineuronal nets (PNNs). PNN formation coincides with the closure of the visual critical period (Sigal et al., 2019) and cleavage of PNN components in adults can partially reopen CP plasticity (Pizzorusso et al., 2002). This process is developmentally regulated by astrocytes, as their progressive increase in the expression of the gap junction connexin 30 inhibits the expression of a PNN degrading enzyme, promoting PNN formation, PV IN synaptic maturation, and closure of the CP (Ribot et al., 2021). In S1, PNN and PV expression increase between P10 and 20, coincident with the closure of the critical period for LIV sensory-dependent plasticity (Nowicka et al., 2009). Moreover, PNN expression increases in sensory-deprived barrels when all but one of the whiskers are trimmed. Therefore, both in V1 and S1, PNNs are considered molecular breaks on synaptic plasticity promoting the closure of CPs. Similarly, Kv3.1 channels, increase their expression level in PV INs during late postnatal development and are necessary for their characteristic fast spiking phenotype (Erisir et al., 1999). Kv3.1 loss of function in PV INs results in a slower rate of input loss in V1, caused by monocular deprivation, although the timing of the critical period remains unchanged (Matsuda et al., 2021). Consistent with this observation, rapid changes in PV inhibitory microcircuits underlie CP plasticity, contributing to circuit reconfiguration in response to sensory deprivation. In S1, a single day of whisker deprivation reduces LIV feedforward inhibition onto LII-III Pyr, by a rapid reduction in PV intrinsic excitability (Gainey et al., 2018). In V1, one day of monocular deprivation reduces the firing rate of LII-III PV INs *in vivo*, due to a reduction in their excitatory drive from LIV-V (Kuhlman et al., 2013). This rapid disinhibition would not only maintain a stable neuronal firing rate in the absence of sensory input, as a homeostatic mechanism but also, could be permissive for Hebbian modes of plasticity to take place, resulting in a reconfiguration of cortical circuits (Gainey and Feldman, 2017). All this evidence shows how manipulation of different factors involved in PV IN function have a direct impact on the properties of the CP, underscoring the pivotal role of this IN subtype in this process.

While the evidence for the role of other IN subtypes in CP plasticity has been limited, a few studies have started to shed light on how other INs can influence the CP indirectly *via* their interaction with PV INs (Figure 3). Transplantation experiments with genetically purified embryonic SST INs in V1 are able to reopen CP plasticity in adults (Tang et al., 2014), as seen with the mixed MGE-derived or purified PV population. Indicating a possible role for SST INs in CP plasticity, likely by a molecular or synaptic interaction with PV INs. Consistent with this idea, another study showed how increased SST activity through the nicotinic modulator Lypd6 can reopen critical period plasticity in adult V1 by means of their inhibitory effect onto PV, resulting in Pyr disinhibition (Sadahiro et al., 2020). In addition, cross-talk between SST and PV INs can influence PV IN function. It was recently shown that SST INs produce Collagen19, which promotes the development of PV perisomatic innervation and removal of this signal reduces anatomical PV innervation and leads to behavioral defects (Su et al., 2020). Therefore, SST interaction with PV, either by synaptic or chemical signaling can influence critical period plasticity. In a similar way, silencing of LI INs results in the abolishment of map plasticity during the tonotopic CP in A1; this effect is mediated by their innervation of LIV Pyr and PV INs and the resulting modulation of thalamic drive onto LIV (Takesian et al., 2018). Beyond the CP, adult visual plasticity is potentiated by locomotion, this effect can be recapitulated upon VIP IN stimulation and involves the VIP-SST disinhibitory circuit (Fu et al., 2015). Further research in this direction will paint a more complete picture and a better understanding on how different elements of cortical circuits influence critical period plasticity.

## Conclusions and Future Directions

Accumulated evidence from studies in cortical development highlight the crucial and diverse roles of inhibitory microcircuits all along early development, critical period and into adulthood. Special attention must be taken into the developmental stage, cortical area, and layer specificity as all of these factors can dictate the function of cortical interneurons in sculpting neuronal activity and proper cortical maturation. Technical advances in *in vivo* imaging, manipulation of neuronal activity and circuit mapping with precise temporal and neuronal subtype resolution will help unravel some of the transient cortical circuits in development that constitute keystones for the normal maturation of sensory cortices. In addition, high throughput single-cell sequencing, can guide the search for molecular factors that can influence neuronal maturation and critical period plasticity with cell-type and temporal specificity (Kalish et al., 2020). These new tools and technological advances can be used to address the mechanisms that regulate other forms of plasticity in S1 and A1, beyond the well characterized ODP in V1. Along the same lines, what constitutes the basis of life-long forms of plasticity in S1, not fully restricted to temporally-bound critical periods as in the other sensory areas, is another question that remains unaddressed.

Multiple neurodevelopmental disorders such as attention-deficit/hyperactivity disorder (ADHD), autism, schizophrenia, among others, display abnormal GABAergic function/connectivity (Ramamoorthi and Lin, 2011). Moreover, PV expression is heavily downregulated in human postmortem autism spectrum disorder (ASD) compared to control samples (Schwede et al., 2018). Suggesting that critical period plasticity in humans could be also be disrupted in some of these disease states as a result of GABAergic and PV IN dysfunction. However, our knowledge of the underlying causes of neurodevelopmental diseases is still limited. In this context two major questions remain: how early developmental disruption of cortical inhibition can result in permanent changes in cortical connectivity? and what are the specific circuits that are affected by disruption of different inhibitory INs subtypes? A better understanding of the basic mechanisms of cortical postnatal development and all the players involved in this process (microcircuits, neuronal subtypes, regulatory mechanisms) will provide cues into neurodevelopmental diseases in which normal cortical maturation is impaired and pave the road for more effective diagnosis, treatment, and disease management. Moreover, our knowledge about development in primary sensory areas, in normal and disease states, can hint and extrapolate to alterations in high order cognitive areas tightly linked to behavioral deficits in neurodevelopmental disorders.

Although rodents are extremely useful experimental models to approach mammalian systems as a first step to understand human biology and disease, there are species-specifc differences in the developmental roadmap, timing or underlying mechanisms in cortical neurodevelopment across species. An example of this is the thalamocortical innervation in humans and other primates that occurs before cortical neurogenesis; in contrast to rodents, in which this occurs by the end of neurogenesis (Alzu’bi et al., 2019). How the timing of thalamocortical innevation can influence cortical development differentially in primates and rodents is unknown. In addition to this, there are difficulties establishing clear equivalents between mouse and human neurodevelopment. In this context, animal studies and human organoid studies can mutually complement and build from each other: on one hand having all the advantages of the mouse tools, including *in vivo* and behavioral analysis; while on the other hand having the ability to use of differentiated human pluripotent cells to recapitulate neurodevelopment in organoids *in vitro* (Birey et al., 2022). These different approaches in concert can give us a better understanding of human normal neurodevelopment and disease states.

## Author Contributions

Both authors conceptualized, contributed and wrote the article. All authors contributed to the article and approved the submitted version.

## Conflict of Interest

The authors declare that the research was conducted in the absence of any commercial or financial relationships that could be construed as a potential conflict of interest.

## Publisher’s Note

All claims expressed in this article are solely those of the authors and do not necessarily represent those of their affiliated organizations, or those of the publisher, the editors and the reviewers. Any product that may be evaluated in this article, or claim that may be made by its manufacturer, is not guaranteed or endorsed by the publisher.
